# Urban brown rats (*Rattus norvegicus*) as possible source of multidrug-resistant Enterobacteriaceae and meticillin-resistant *Staphylococcus* spp., Vienna, Austria, 2016 and 2017

**DOI:** 10.2807/1560-7917.ES.2019.24.32.1900149

**Published:** 2019-08-08

**Authors:** Amélie Desvars-Larrive, Werner Ruppitsch, Sarah Lepuschitz, Michael P Szostak, Joachim Spergser, Andrea T Feßler, Stefan Schwarz, Stefan Monecke, Ralf Ehricht, Chris Walzer, Igor Loncaric

**Affiliations:** 1 University of Veterinary Medicine, Vienna, Austria; 2 Austrian Agency for Health and Food Safety, Vienna, Austria; 3 Freie Universität, Berlin, Germany; 4 Leibniz Institute of Photonic Technology (IPHT), Jena, Germany; 5 Technische Universität, Dresden, Germany; 6 InfectoGnostics Research Campus, Jena, Germany; 7 Wildlife Conservation Society, Bronx, New York, United States

**Keywords:** Rattus norvegicus, antimicrobial resistance, Enterobacter xiangfangensis ST114, *Escherichia coli*, meticillin-resistant Staphylococcus, NDM-1, ESBL

## Abstract

**Background:**

Brown rats (*Rattus norvegicus*) are an important wildlife species in cities, where they live in close proximity to humans. However, few studies have investigated their role as reservoir of antimicrobial-resistant bacteria.

**Aim:**

We intended to determine whether urban rats at two highly frequented sites in Vienna, Austria, carry extended-spectrum β-lactamase-producing Enterobacteriaceae*,* fluoroquinolone-resistant Enterobacteriaceae and meticillin-resistant (MR) *Staphylococcus* spp. (MRS).

**Methods:**

We surveyed the presence of antimicrobial resistance in 62 urban brown rats captured in 2016 and 2017 in Vienna, Austria. Intestinal and nasopharyngeal samples were cultured on selective media. We characterised the isolates and their antimicrobial properties using microbiological and genetic methods including disk diffusion, microarray analysis, sequencing, and detection and characterisation of plasmids.

**Results:**

Eight multidrug-resistant *Escherichia coli* and two extensively drug-resistant New Delhi metallo-β-lactamases-1 (NDM-1)-producing *Enterobacter xiangfangensis* ST114 (*En. cloacae* complex) were isolated from nine of 62 rats. Nine Enterobacteriaceae isolates harboured the *bla*_CTX-M_ gene and one carried a plasmid-encoded *ampC* gene (*bla*_CMY-2_). Forty-four MRS were isolated from 37 rats; they belonged to seven different staphylococcal species: *S. fleurettii*, *S. sciuri*, *S. aureus*, *S. pseudintermedius*, *S. epidermidis*, *S. haemolyticus* (all *mecA*-positive) and *mecC*-positive *S. xylosus*.

**Conclusion:**

Our findings suggest that brown rats in cities are a potential source of multidrug-resistant bacteria, including carbapenem-resistant *En*. *xiangfangensis* ST114. Considering the increasing worldwide urbanisation, rodent control remains an important priority for health in modern cities.

## Introduction

The emergence of multidrug-resistant pathogens that are difficult, and sometimes impossible, to treat is becoming a global concern for human and domestic animal health [1,2]. Extended-spectrum β-lactamase (ESBL)-producing Enterobacteriaceae (ESBL-E), carbapenem-resistant Enterobacteriaceae (CRE) and meticillin-resistant (MR) *Staphylococcus aureus* (MRSA) are recognised as a threat to healthcare and patient safety [1]. One of the most recently described carbapenemase genes, the New Delhi metallo-β-lactamase-1 (NDM-1) gene, is located on self-transmissible plasmids that carry several additional antimicrobial resistance genes, which makes NDM-1 a challenge for public health [3]. The role of urban wildlife as reservoirs and/or vectors of antimicrobial resistance (AMR) is poorly understood. Not only do they provide a biological mechanism for the spread of AMR genes [4], they are also considered sentinels of environmental pollution by antimicrobial-resistant bacteria [5].

More than half of the world population now is urban and by 2030, that fraction will have increased to 60% [6]. Cities can serve as focal points for pathogen introduction and dissemination [7]. The dense human population, increased interactions with urban wildlife and the warmer urban microclimate favour the emergence of wildlife-borne zoonoses in cities [7]. With regard to AMR, wild brown rats (*Rattus norvegicu*s) are particularly relevant. Rats are considered the most prolific and widespread urban pest species [8]. Synanthropic rats, foraging on human waste and colonising the sewage system, frequently interact with human faeces. Therefore, they are exposed to anthropogenic antimicrobial residues and can acquire, carry and spread multidrug-resistant bacteria [9,10]. However, little is known about their role in the epidemiology of AMR. Antimicrobial-resistant Enterobacteriaceae were isolated from urban rats in Piraeus, Greece [11], Berlin, Germany [9], Hong Kong [12] and Vancouver, Canada [13]. Kato et al. reported carriage of antimicrobial-resistant *S. aureus* in urban rats in Tokyo, Japan, although meticillin resistance was not mentioned [14]. Prevalence of MRSA and MR *Staphylococcus pseudintermedius* (MRSP) in urban rats was investigated in Vancouver, Canada [15,16] while Hansen et al. described the resistome of urban brown rats in Malaysia, Hong Kong, and Denmark [10].

Because each city presents a specific environmental and socioeconomic context, local studies are needed to evaluate the role of urban rats in the epidemiology of AMR. Our main objective was to determine whether urban rats from two highly frequented places of the city centre of Vienna, Austria, carry ESBL-E, fluoroquinolone-resistant Enterobacteriaceae and MR *Staphylococcus* spp (MRS).

## Methods

### Sampling protocol

*Rattus norvegicus* were trapped on 17 occasions between 12 September 2016 and 6 June 2017 in the city centre of Vienna, Austria: (i) at Karlsplatz (16.37124, 48.20035 decimal degrees (D.D.)), one of the most touristic places in the city, and (ii) at a promenade along the Danube Canal (48.22631, 16.36541 D.D.). These places were investigated because rats were observed during daytime (whereas *R. norvegicus* is mostly described as a nocturnal species [8]), which suggested an abundant rat population, and because high densities of rats, humans and companion animals may increase contact rates and the risk of pathogen transmission [17].

Rats were captured alive using Manufrance live-traps (280 × 100 × 100 mm). At each location, 20 to 60 traps per night (Karlsplatz: 10 nights, Danube Canal: seven nights) were set between 17.00 and 19.30 and retrieved between 6.00 and 8.00. The trapping effort per site was adjusted according to the method described by Nelson and Clark [18]. Captured animals were transferred alive to the pathology laboratory where morphometric data and samples were collected. Rats were anesthetised using 5% isoflurane before euthanasia through barbiturate overdose via the intra-peritoneal route. We collected pharyngeal and deep intra-nasal samples using sterile cotton swabs. Swabs were placed individually in Amies transport medium (Heinz Herenz Medizinalbedarf GmbH, Hamburg, Germany) until culture. During necropsy, small intestine and colon tissues were collected aseptically. Freshly collected samples were transported to the Institute of Microbiology in a cooled box.

### Characterisation of Enterobacteriaceae isolates

#### β-lactamase-producing Enterobacteriaceae

For each animal, pooled intestinal tissue samples (small intestine and colon) were precultured in buffered peptone water (Merck, Darmstadt, Germany) supplemented with 1 mg/L cefotaxime and cultivated at 37 °C overnight on MacConkey agar (Oxoid, Basingstoke, United Kingdom (UK)) supplemented with 1 mg/L cefotaxime.

#### Fluoroquinolone-resistant Enterobacteriaceae

In parallel, intestinal samples were cultivated at 37 °C overnight on MacConkey Broth (Becton Dickinson, Heidelberg, Germany) and cultivated on MacConkey agar supplemented with 0.06 mg/L ciprofloxacin. For each plate, a single sample of each distinct colony type was picked and regrown on the same medium.

The Enterobacteriaceae isolates were identified by matrix-assisted laser desorption/ionisation-time-of-flight (MALDI-TOF) mass spectrometry (Bruker Daltonik, Heidelberg, Germany). We tested the enterobacterial isolates for production of ESBL using combination disk tests containing cefotaxime and ceftazidime, with or without clavulanic acid (Becton Dickinson, Heidelberg, Germany), according to the Clinical and Laboratory Standards Institute (CLSI) [19]. Furthermore, we used disks containing 30 μg cefoxitin and 5 μg ciprofloxacin (Becton Dickinson, Heidelberg, Germany) to screen for, respectively, ampicillin C (AmpC) β-lactamase-producing and fluoroquinolone-resistant isolates. The ESBL and AmpC phenotypes were confirmed by MASTDISCS ID AmpC and ESBL test (Mast Diagnostics, Merseyside, UK).

We tested the susceptibility of the Enterobacteriaceae isolates against selected antimicrobial agents by the agar disk diffusion method according to the CLSI guidelines [19]. *Escherichia coli* ATCC 25922 and *S. aureus* ATCC 25923 served as quality control strains.

We characterised resistance genes using a miniaturised microarray-based assay (CarbDetect-AS-2 Kit, Alere, Jena, Germany). We performed serogenotyping and detected resistance and major virulence genes of *E. coli* isolates using the *E. coli* PanType AS-2 kit (Alere, Jena, Germany). We used PCR to detect *bla* and *qnr* genes and then sequenced the amplified products [20,21]. Sequences were aligned with BLAST (https://blast.ncbi.nlm.nih.gov/Blast.cgi) and compared with reference sequences available in GenBank and the National Center for Biotechnology Information (NCBI) database (http://www.ncbi.nlm.nih.gov/pathogens/beta-lactamase-data-resources/). Variable regions of class 1 and class 2 integrons were determined by PCR [22]. The quinolone resistance-determining regions (QRDR) of *gyrA* and *parC* in ciprofloxacin-resistant isolates were amplified by PCR and sequenced [23]. Using PCR and sequencing, we investigated the genetic environment of *bla*_CTX-M_ genes, i.e. the upstream insertion sequences (IS)*Ecp1* and IS*26* involved in gene-transferring mechanisms [22,24].

The *E. coli* isolate were assigned to a phylogroup using the quadruplex assignment method [25] and were further subjected to multilocus sequence typing (MLST) [26]. Allelic profiles and sequence types (ST) were determined by querying the *E. coli* MLST website (http://enterobase.warwick.ac.uk/species/ecoli/allele_st_search). We performed MLST of the *Enterobacter cloacae* complex according to the protocol recommended by Miyoshi-Akiyama et al. [27]. 

We assessed the clonal relatedness of two *bla*_NDM-1_-positive isolates from the *En. cloacae* complex by whole genome sequencing (WGS). Bacterial DNA was isolated using the MagAttract HMW DNA Kit (Qiagen, Hilden, Germany) and ready-to-sequence libraries were prepared using Nextera XT DNA Library Preparation Kit (Illumina, San Diego, United States). Isolates were paired-end-sequenced using the Illumina MiSeq platform with a read length of 2 × 300 bp [28]. De novo assembly of raw reads was performed using SPAdes v.3.9.0 [29] and species identification was conducted with the JSpecies workspace using the ANIb (average nucleotide identity via BLAST) analysis tool [30]. We used the Comprehensive Antibiotic Resistance Database (CARD; https://card.mcmaster.ca/home) to identify genes conferring AMR [31].

The presence of plasmids was determined using PlasmidFinder 1.3 available from the Center for Genomic Epidemiology web server (http://www.genomicepidemiology.org/) [32]. We performed conjugation experiments with *bla* gene-carrying isolates as donors, confirmed and characterised the transconjugants as previously described [20]. We conducted electrotransformation experiments using a GenePulser II (BioRAD, Vienna, Austria). The conjugative and transferable plasmids were subjected to PCR-based replicon typing [33].

### Characterisation of *Staphylococcus* spp. isolates

For each animal, pharyngeal and nasal samples were pooled [34], incubated overnight in tryptic soy broth (Becton Dickinson, Heidelberg, Germany) medium with 6.5% NaCl, then streaked on a BBL CHROMagar MRSA II (Becton Dickinson, Heidelberg, Germany) and Mueller-Hinton agar (Oxoid, Basingstoke, UK) with 2.5% NaCl, 2 mg/L oxacillin and 20 mg/L aztreonam. For each plate, a single sample of each distinct colony type was picked and regrown on the same medium. Meticillin resistance was confirmed by an agar disk diffusion method with 30 µg cefoxitin or 1 µg oxacillin (Becton Dickinson, Heidelberg, Germany) [19].

The *mecA* or *mecC* genes were amplified by PCR [35] and MRS were characterised to species level by PCR amplification and sequencing of the *rpoB* gene [36]. Those MR staphylococci harbouring *mecA* or *mecC* were tested for susceptibility against selected antimicrobial agents using the agar disk diffusion method according to the CLSI guidelines [19]. Detection of resistance and virulence genes was conducted using the *S. aureus* Genotyping Kit 2.0 DNA microarray (Alere, Jena, Germany) [37]. We further analysed MRSA, MRSP, MR *S. epidermidis* (MRSE) and MR *S. haemolyticus* (MRSH) by *dru*-typing, while MRSA and MRSP were additionally examined by *spa*-typing [38]. We analysed the staphylococcal cassette chromosome *mec* (SCC*mec*) elements of some selected isolates using a DNA-based-microarray (Alere, Jena, Germany) [39].

**Figure 1:**
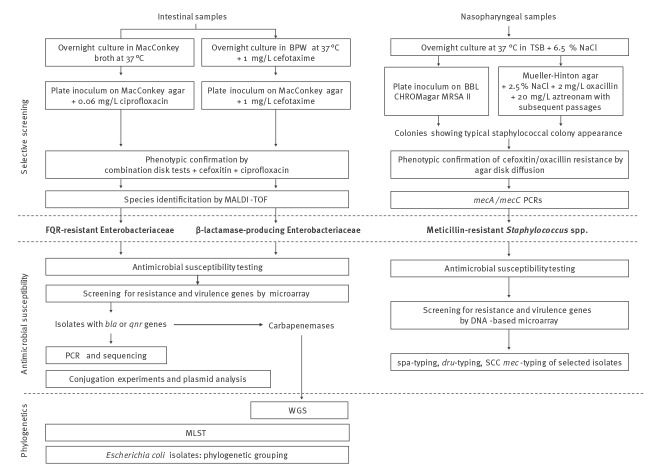
Workflow of the laboratory testing of urban brown rats for antimicrobial-resistant bacteria, Vienna, 13 September 2016–6 June 2017

BPW: buffered peptone water; FQR: fluoroquinolone; MALDI-TOF: matrix-assisted laser desorption/ionisation-time-of-flight mass spectrometry; MLST: multilocus sequence typing; MRSA: meticillin-resistant *Staphylococcus* *aureus*; TSB: tryptic soy broth; WGS: whole genome sequencing.

A workflow of the laboratory procedures is provided in Figure 1.

### Statistical analyses

We considered the following explanatory variables: *place of capture*, *body mass* (g), *body length* (nose to anus, mm) and *sex*. The impact of these factors on the occurrence of AMR (presence of at least one resistant isolate) and on the number of antimicrobial-resistant isolates per animal was assessed using, respectively, a generalised linear model under a binomial and a Poisson distribution. The best-fitted model was identified using a stepwise backward selection based on the Akaike Information Criterion. Statistical analyses were conducted using R 3.5.1 (R Development Core Team, Vienna, Austria).

### Nucleotide sequence accession numbers

Because the two *En.* *xiangfangensis* isolates are most probably emerging in Austria, it is of public health importance to make their sequences publicly available. Therefore, the genomes of these two isolates were deposited under PRJNA517386 in the NCBI BioProject database.

### Ethical statement

We followed institutional and national standards for the care and use of animals in research. This study was approved by the institutional ethics and animal welfare committee and the national authority (GZ 68.205/0196-WF/V/3b/2016).

## Results

### Trapping

Seventy-six brown rats were captured. Among them, 14 were not further analysed because samples could not be sent fast enough to the Institute of Microbiology after necropsy. Thirty-six rats from Karlsplatz (of which 18 were male) and 26 from Danube Canal (15 males) were ultimately included in the study (Supplementary Figure S1). The median body weight and length were 193.5 g and 196.5 mm on Karlsplatz, 153.2 g and 184.0 mm at Danube Canal (Supplementary Table S1). The adjusted trapping effort was 284.5 trap-nights at Karlsplatz and 238 at Danube Canal.

### Characterisation of Enterobacteriaceae isolates

Ten cefotaxime-resistant Enterobacteriaceae were isolated from the intestinal samples of nine of the 62 rats (Table 1). We identified eight isolates as *E. coli* and two as members of the *En. cloacae* complex. Seven serogenotypes were detected among the *E. coli* isolates. All 10 isolates were susceptible to amikacin. Seven *E. coli* isolates displayed the ESBL phenotype. One *E. coli* isolate and both isolates from the *En. cloacae* complex displayed the AmpC and ESBL phenotype. All Enterobacteriaceae isolates were ampicillin- and cefazolin-resistant, while four *E. coli* and both isolates from the *En. cloacae* complex were also tetracycline- and fluoroquinolone-resistant. Both isolates from the *En. cloacae* complex were carbapenem-resistant. Based on Sweeney et al., these two isolates can be considered as extensively drug-resistant (not susceptible to at least one agent in all but one or two available antimicrobial classes) [40].

**Table 1:** Molecular characteristics of Enterobacteriaceae isolates from wild urban brown rats, Vienna, Austria, 13 September 2016–6 June 2017 (n = 10)

Isolate^a^	Species	Resistance profile	Serogenotyping		Phylogroup	ST	Antimicrobial resistance genes				Virulence factors	CTX environment			Repli-con	Transconjugants
			O-serotyping	H-serotyping			β-lactamase	Non-β-lactamase	Mutation in *gyrA*	Mutation in *parC*		IS*Ecp1* 5´	IS*Ecp1* UP	*tnpA* IS26		
AD8E	*Escherichia coli*	AMP, CFZ, CTX, FQR	21	21	B1	ST101	*bla*_CMY-2_, *bla*_TEM-1_	NA	83Ser-Leu, 87Asp-Asn	80Ser-Ile	*hemL, iss*	NA	NA	NA	NA	NA
AD10Ea	*E. coli*	AMP, CFZ, CTX, FQR, TET, SXT	ND	33	C	ST34	*bla*_CTX-M-15_, *bla*_TEM-1_	*aadA1, dfrA1, qrnS, sul2, tet*(A)	83Ser-Val	Wild type	*hemL, intI1, mchB, mchC, mchF, pic, sat, sepA*	1.7	0.25	NA	NA	NA
AD10Eb	*E. coli*	AMP, CFZ, CTX, TET, SXT	86	18	E	ST38	*bla*_CTX-M-9_	*strB, sul2*	Wild type	Wild type	*eatA, hemL, iss, nfaE*	NA	NA	NA	NA	NA
AD19E	*E. coli*	AMP, CFZ, CTX	6	49	B1	ST1079	*bla*_CTX-M-3_	NA	NA	NA	NA	NA	0.25	NA	NA	NA
AD21E	*E. coli*	AMP, CFZ, CTX	6	49	B1	ST1079	*bla*_CTX-M-3_	NA	NA	NA	NA	NA	0.25		F	*bla*_CTX-M-3_
AD24E	*E. coli*	AMP, CFZ, CTX, SXT	8	25	B1	ST58	*bla*_CTX-M-9,_ *bla*_TEM-176_	*dfrA5, sul2, strB*	NA	NA	*prfB, ireA, iss*	NA	NA	NA	NA	NA
AD32E	*E. coli*	AMP, CFZ, CTX, ATM, FQR, TET, CHL, SXT	9	30	C	ST361	*bla*_CTX-M-15,_ *bla*_TEM-176_	*aadA4, aphA, dfrA17,strB, catA1, sul2, tet*(B)	83Ser-Leu, 87Asp-Asn	80Ser-Ile	*iss*	2.5	0.25	NA	FIB, F	*bla*_CTX-M-15,_ *bla*_TEM-176_
AD54E	*Enterobacter xiangfangensis*	AMP, CFZ, CTX, FEP, IPM, MEM, ETP, ATM, FQR, GEN, TOB, TET, CHL, SXT	NA	NA	NA	ST114	*bla*_NDM-1_, *bla*_CTX-M-15,_ *bla*_TEM176,_ *bla*_OXA-1_	*aac(6')-Ib-cr, aadA1, aadA2, catA1, catB3, dfrA1, dfrA12, dfrA14, qrnB1, ramA, sul1*	83Ser-Leu	NA	NA	2.5	0.25	NA	H12	*bla*_CTX-M-15_, *bla*_TEM-176_, *bla*_OXA-1_, *aac(6')-Ib, aadA1, aadA2, catA1, catB3, dfrA1, dfrA12, dfrA14, qrnB1, sul1*
AD72E	*En. xiangfangensis*	AMP, CFZ, CTX, FEP, IPM, MEM, ETP, ATM, FQR, GEN, TOB, TET, CHL, SXT	NA	NA	NA	ST114	*bla*_NDM-1_, *bla*_CTX-M-15,_ *bla*_TEM176,_ *bla*_OXA-1_	*aac(6')-Ib-cr, aadA1, aadA2, catA1, catB3, dfrA1, dfrA12, dfrA14,qrnB1, ramA, sul1*	83Ser-Leu	NA	NA	2.5	0.25	NA	H12	*bla*_CTX-M-15_, *bla*_TEM-176_, *bla*_OXA-1,_ *aac(6')-Ib, aadA1, aadA2, bla_CTX_, bla_TEM_, bla_OXA_, catA1, catB3, dfrA1, dfrA12, dfrA14, qrnB1, sul1,*
AD74E	*E. coli*	AMP, CFZ, CTX, ATM, FQR, TET, CHL, SXT	8	30	B1	ST1431	*bla*_CTX-M-1,_ *bla*_TEM-1_	*aadA1,aadA2, aphA, tet*(A), *catA1, cmlA1, dfrA12, floR, sul2, sul3*	83Ser-Leu, 87Asp-Asn	80Ser-Ile	*hemL, intI1, iss*	> 3	3	NA	N	*bla*_CTX-M-1,_ *bla*_TEM-1_

AMP: ampicillin; ATM: aztreonam; CFZ: cefazolin; CHL: chloramphenicol; CTX: cefotaxime; ETP: ertapenem; FEP: cefepime; FQR: fluoroquinolone; GEN: gentamicin; IPM: imipenem; MEM: meropenem; NA: data not available; ND: not detected; ST: sequence type; SXT: trimethoprim-sulfamethoxazole; TET: tetracycline; TOB: tobramycin.

^a^ Rat ID + E: Enterobacteriaceae isolate. When several isolates were identified, they were given a letter (a, b, etc).

The *E. coli* isolates belonged to three different phylogroups: B1, C and E. Seven different *E. coli* ST were detected. MLST identified both members of the *En. cloacae* complex as ST114. WGS-based ANIb analysis identified these two isolates as *En. xiangfangensis* carrying the NDM-1 gene on a plasmid similar to type IncH (GenBank accession number: CP016921). PlasmidFinder identified this plasmid as belonging to the IncHI2 group.

In nine of 10 *E. coli* isolates, genes from the *bla*_CTX-M_ family were detected, alone (three isolates) or in combination with other *bla* genes (four isolates). The *E. coli* AD8E isolate harboured the genes *bla*_CMY-2_ and *bla*_TEM-1_. Both *En. xiangfangensis* isolates carried four *bla* genes (*bla*_NDM-1_, *bla*_CTX-M-15_, *bla*_TEM-176_, and *bla*_OXA-1_).

The genes *sul2* and *aadA1* were the most prevalent non-β-lactamase genes detected. A class 1 integron was detected in four ESBL-producing *E. coli* and in both *En. xiangfangensis* isolates. However, we could not amplify the variable regions of the class 1 integron by PCR. Amino acid substitutions were detected in the QRDR of *gyrA* and *parC*. Complete or truncated IS*Ecp1* elements were detected upstream of the *bla*_CTX-M_ gene in seven isolates, five *E. coli* and both *En. xiangfangensis*. We did not identify the IS*26* element in any isolate. The CTX-M-positive isolates showed a 48-bp region W sequence upstream of gene *bla*_CTX-M-1_, as previously described (GenBank accession number: AM040707), whereas one CTX-M-1-positive isolate harboured the same 48-bp W sequence plus a 32-bp X sequence, as previously described (GenBank accession number: AM003904).

The *hemL* gene (encoding the glutamate-1-semialdehyde aminotransferase) and the *iss* (increased serum survival) gene were the most prevalent virulence determinants among the *E. coli* isolates. Transfer of resistance was demonstrated for *bla* genes by conjugation of three ESBL-producing *E. coli* isolates as donors with either the sodium azide-resistant *E. coli* J53 or the sodium azide- and rifampin-resistant *E. coli* MT 102 as the recipient. Using both *En. xiangfangensis* isolates as donors, conjugation and transformation of the *bla*_NDM-1_ gene were unsuccessful but transfer of the *bla*_CTX-M_ genes was achieved.

### Characterisation of meticillin-resistant *Staphylococcus* spp. 

Forty-four MRS belonging to seven species were isolated from 37 of 62 rats (Table 2). Among them, we identified MRSA (n = 1; prevalence in the rat population: 1.6%), MRSP (n = 1), MRSE (n = 1), MRSH (n = 1), MR *S. xylosus* (n = 1) and two species from the *S. sciuri* group, i.e. MR *S. fleurettii* (n = 37 isolates; 54.8%) and MR *S. sciuri* (n = 2; 3.2%). All MRS were *mecA*-positive with the exception of the single MR *S. xylosus* that was *mecC*-positive. All MR *S. fleurettii* and the MR *S. xylosus* were susceptible to all tested non-β-lactam antimicrobial agents. The other six MR staphylococcal isolates were classified as multidrug-resistant [40].

**Table 2:** Resistance and virulence genetic profile of *Staphylococcus* spp. isolates from wild urban brown rats, Vienna, Austria, 13 September 2016–6 June 2017 (n = 44)

Isolate^a^	Species^b^	No. isolates	*dru*	SCC*mec*	CC	Antimicrobial resistance profile		Virulence factors
						Phenotype	Genes detected	
AD6	*S. fleurettii*	19	NA	Irregular	NA	BLA	*mecA*	NA
AD7	*S. fleurettii*	19	NA	Irregular	NA	BLA	*mecA*	NA
AD8	*S. fleurettii*	19	NA	Irregular	NA	BLA	*mecA*	NA
AD9	*S. fleurettii*	19	NA	Irregular	NA	BLA	*mecA*	NA
AD9b_MRSP	*S. pseudintermedius*	1	dt11av	V	NA	BLA, TET, ERY, CLI, AMK, GEN, FQR	*mecA, bla*Z, *blaI, blaR, tet*(K), *tet*(M), *erm*(B), *aacA-aphD, aphA3, sat*	*eno, isdA, lukS *(ST22 + ST45)
AD10	*S. fleurettii* (2)^c^	1	NA	Irregular	NA	BLA	*mecA*	NA
AD10b_S.xylosus_mecC	*S. xylosus* ^c^	1	NA	XI	NA	BLA	*mecC, blaZ-SCCmec* XI	NA
AD11	*S. fleurettii* (5)^c^	1	NA	Irregular	NA	BLA	*mecA*	NA
AD13	*S. fleurettii* (6)^c^	1	NA	Irregular	NA	BLA	*mecA*	NA
AD15	*S. fleurettii* (7)^c^	1	NA	Irregular	NA	BLA	*mecA*	NA
AD16	*S. fleurettii*	3	NA	Irregular	NA	BLA	*mecA*	*ssl10/set4*
AD16	*S. fleurettii* (11)^c^	1	NA	Irregular	NA	BLA	*mecA*	*ssl10/set4*
AD17	*S. fleurettii*	3	NA	Irregular	NA	BLA	*mecA*	*ssl10/set4*
AD19	*S. sciuri*	2	NA	Irregular	NA	BLA, ERY, TET	*mecA, blaZ, blaI, blaR, tet*(M), *erm*(B)	NA
AD23	*S. fleurettii*	3	NA	Irregular	NA	BLA	*mecA*	*ssl10/set4*
AD24	*S. fleurettii*	19	NA	Irregular	NA	BLA	*mecA*	NA
AD25	*S. fleurettii*	19	NA	Irregular	NA	BLA	*mecA*	NA
AD27	*S. fleurettii* (8)^c^	1	NA	Irregular	NA	BLA	*mecA*	NA
AD28	*S. fleurettii* (9)^c^	1	NA	Irregular	NA	BLA	*mecA*	NA
AD30	*S. fleurettii*	19	NA	Irregular	NA	BLA	*mecA*	NA
AD31	*S. fleurettii*	19	NA	Irregular	NA	BLA	*mecA*	NA
AD32	*S. fleurettii*	19	NA	Irregular	NA	BLA	*mecA*	NA
AD33	*S. fleurettii* (10)^c^	1	NA	Irregular	NA	BLA	*mecA*	NA
AD34	*S. fleurettii*	1	NA	Irregular	NA	BLA	*mecA*	*isaB, isdA, lukS *(ST22 + ST45)
AD35	*S. fleurettii*	19	NA	Irregular	NA	BLA	*mecA*	NA
AD36	*S. fleurettii* (12)^c^	1	NA	Irregular	NA	BLA	*mecA*	NA
AD37	*S. fleurettii* (13)^c^	1	NA	Irregular	NA	BLA	*mecA*	NA
AD37	*S. fleurettii* (4)^c^	1	NA	Irregular	NA	BLA	*mecA*	*hlIII*
AD38	*S. fleurettii*	19	NA	Irregular	NA	BLA	*mecA*	NA
AD39	*S. fleurettii*	19	NA	Irregular	NA	BLA	*mecA*	NA
AD50	*S. sciuri*	2	NA	Irregular	NA	BLA, ERY, TET	*mecA, blaZ, blaI, blaR, tet*(M), *erm*(B)	NA
AD52	*S. fleurettii*	19	NA	Irregular	NA	BLA	*mecA*	NA
AD59	*S. fleurettii*	1	NA	Irregular	NA	BLA	*mecA*	*isaB, isdA, lukS *(ST22 + ST45*), ssl10/set4*
AD61	*S. fleurettii* (3)^c^	1	NA	Irregular	NA	BLA	*mecA*	*ssl10/set4*
AD68_MRSE	*S. epidermidis*	1	dt10a	Pseudo SCC*mec*	NA	BLA, TET, ERY, CLI, AMK, GEN	*mecA, bla*Z, *blaI, blaR, tet*(K) *erm*(C), *dfrA, aacA-aphD*	NA
AD69a	*S. fleurettii*	19	NA	Irregular	NA	BLA	*mecA*	NA
AD69b	*S. fleurettii*	19	NA	Irregular	NA	BLA	*mecA*	NA
AD70	*S. fleurettii* (1)^c^	1	NA	Irregular	NA	BLA	*mecA*	*ebpS, isaB, isdA, lukS *(ST22 + ST45)
AD70_MRSA	*S. aureus* ^c^	1	dt11a	VT	CC398	BLA, TET, ERY, CLI	*mecA, bla*Z, *tet*(K), *tet*(M), *erm*(A)	*aur, bbp,cap5, capH5, capJ5, capK5, clfA, clfB, cna, edh, ebpS, emp, fib *(MRSA 252)*, fnbA, fnbB, hl, hla, hlgA, lukF, lukS, hlgA, hsdSx-CC15, hysA1 *(MRSA252 + RF122) and/or* hysA2 (cons), hysA1 *(MRSA252 + RF122) and/or* hysA2 *(COL + US300),* hysA2 *(COL + US300 + NCTC),* icaA, icaC, icaD, isaB *(MRSA 252)* isdA, ImrP, lukX, lukY, map, sdrC, sdrD, setB1, setB2, stB3, setC, set6-var, set11/set2, ssl02/set7, ssl04/set9, ssl05/set3, ssl07/set1, ssl09/set5, ssl10/set4, sspA, sspB, sspP, vwb*
AD71	*S. fleurettii*	19	NA	Irregular	NA	BLA	*mecA*	NA
AD72	*S. fleurettii*	19	NA	Irregular	NA	BLA	*mecA*	NA
AD74	*S. fleurettii*	19	NA	Irregular	NA	BLA	*mecA*	NA
AD74_MRSH	*S. haemolyticus*	1	NA	V	NA	BLA, TET, ERY, CLI, GEN	*mecA, bla*Z, *blaI, blaR, aacA-aphD, mph*(C), *msr*(A), *aadD, aphA3, sat, qacA*	*isdA, ImrP*-variant *sspP-*variant
AD76	*S. fleurettii*	19	NA	Irregular	NA	BLA	*mecA*	NA

AMK: amikacin; BLA: β-lactams; CLI: clindamycin; ERY: erythromycin; FQR: fluoroquinolone; GEN: gentamicin; NA: data not available (absent, therefore not amplified, or not typeable); TET: tetracycline.

^a^ Rat ID + isolate identification if several isolates were isolated from the same rat.

**Figure 2:**
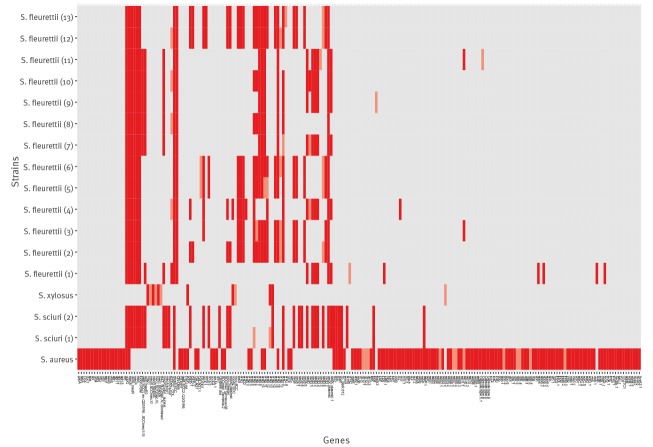
SCC*mec* type of selected MR *Staphylococcus* spp. isolates from wild urban brown rats, Vienna, Austria, 13 September 2016–6 June 2017 (n = 17)

^b^ Number in bracket refers to the isolate identification in Figure 2.

^c^ SCC*mec*-typing via array was performed on these isolates.

The MRSA isolate belonged to the clonal complex 398. It belonged to *spa* type t011, *dru* type dt11a, and was positive for α- and δ-haemolysin, accessory gene regulator (*agr*) group I and capsule type 5. Its SCC*mec* type was SCC*mec* VT + *czrC* as in strain MRSA ST398/isolate S0385 (GenBank accession number: AM990992.1). The MRSP isolate showed *spa* type t02 and *dru* type dt11av. The MRSH isolate belonged to SCC*mec* type V but the *dru* sequence could not be amplified. The MRSE isolate belonged to *dru* type dt10a and had pseudo SCC*mec*. Additional SCC*mec*-typing revealed that members of *S. sciuri* group carried different irregular SCC*mec* elements. The *mecC*-positive MR *S. xylosus* isolate carried the type E *mec* gene complex. The results from SCC*mec* subtyping are presented in Figure 2.

Grey: negative; red: positive; light red: ambiguous.

### Epidemiology of antimicrobial resistance

**Figure 3:**
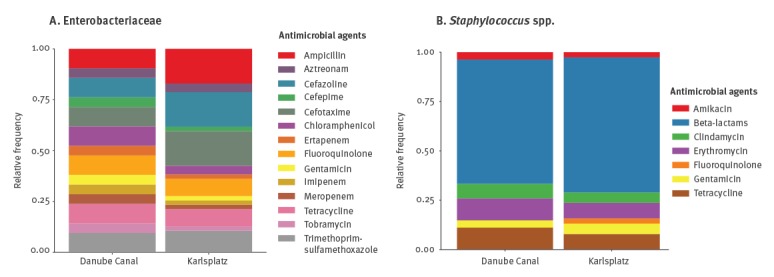
Relative frequencies of antimicrobial resistance phenotypes at each investigated site in (A) Enterobacteriaceae and (B) *Staphylococcus* spp. isolates, Vienna, Austria, 13 September 2016–6 June 2017 (n = 44)

The overall prevalence of antimicrobial-resistant bacteria in the sampled rats was 62.9% (39/62). Twelve of the 39 positive rats showed co-colonisation with two (10 rats), three (1 rat) or four (1 rat) antimicrobial-resistant isolates (Supplementary Table S1, Supplementary Figure S1). Seven Enterobacteriaceae-positive rats were also positive for MRS. Enterobacteriaceae (Figure 3A) and *Staphylococcus* spp. (Figure 3B) isolates from both investigated places shared respectively the same AMR profile, although the relative phenotype frequencies were slightly different and resistance to fluoroquinolone in *Staphylococcus* spp. was only found at Karlsplatz.

These graphs represent the relative frequency (proportion between 0 and 1) of AMR phenotype. The sum of the relative frequencies of all AMR phenotypes per site for each bacterial group is 1.0.

Body weight and length were highly correlated (Spearman’s Rho = 0.96; p < 10^−16^). Therefore, body length was excluded from the models. Occurrence of AMR and number of antimicrobial-resistant isolates per animal were best explained by the single variable *body mass* although the p values of the coefficient estimates were not significant (p = 0.07 and p = 0.11, respectively).

## Discussion

We screened 62 urban brown rats captured in two highly frequented places in the city centre of Vienna, Austria, in 2016 and 2017. We isolated eight multidrug-resistant *E. coli,* two extensively drug-resistant NDM-1-producing *En. xiangfangensis* ST114 and 44 MRS belonging to seven species. To the best of our knowledge, this is the first isolation of NDM-1-producing strains from the *En. cloacae* complex in Austria, although NDM-1 was described in the *En cloacae* complex in other countries [41]. The isolate was identified as *En. xiangfangensis* harbouring simultaneously *bla*_NDM-1_, *bla*_CTX-M-15_, a *bla*_TEM_-like gene, the plasmid-mediated quinolone resistance gene *qrnB1,* and diverse other resistance genes. Moreover, this is the first report of MRSE, MRSH, MR *mecC*-positive *S. xylosus* and MR members of the *S. sciuri* group in synanthropic brown rats.

The prevalence of multidrug-resistant Enterobacteriaceae in urban brown rats in Vienna, Austria (14.5% of 62) was similar to the prevalence reported in Berlin, Germany, (13.6%; sampling period 2008–09) [9] and Hong Kong (13.9%; 2008–13) [12]. In contrast, in Piraeus, Greece, 61.5% of the urban rats carried multidrug-resistant *E. coli* strains (sampling period not specified) [11] while in Vancouver, Canada, the prevalence was 6.5% (2011–12) [13]. All Viennese Enterobacteriaceae isolates were resistant to extended-spectrum cephalosporins, with nine of 10 carrying a *bla*_CTX-M_ (ESBL) gene and one a *bla*_CMY-2_ (AmpC) gene. The spread of these genes is strongly facilitated by plasmid-mediated horizontal gene transfer [42]. These findings suggest that rats may play a role in the dissemination of ESBL and AmpC-type β-lactamases between the human, animal and environmental reservoirs.

To date, two NDM-1-producing *Klebsiella pneumoniae* have been isolated in Austria, from patients originating from Pakistan and Kosovo*, and one NDM-1-producing *E. coli* was isolated from a patient originating from India [43]. Species of the *En. cloacae* complex have recently emerged as the cause of nosocomial outbreaks and difficult-to-treat bacterial infections in the community, and ST114 clones are considered as clones of high epidemic potential [44]. The early detection of emerging carbapenem resistance in urban wildlife is essential in the surveillance and prevention of AMR outbreaks.

We report a high prevalence of MRS in urban brown rats (59.7% of 62) together with a high diversity of MR staphylococcal species. MRSA was isolated in 22 of 637 (3.5%) urban brown rats sampled in 2011–12 in Vancouver, Canada [15], which supports the low MRSA prevalence reported in our study (1.2%). We characterised one isolate as MRSA CC398 VT *spa* type t011, *dru* type dt11a. MRSA CC398 was also identified in wild urban brown rats in Vancouver, Canada, but these strains were slightly different, presenting the *spa* type t034 [15] which only differs from t011 in the duplication of two repeats (t011: 08–16–02–25–34–24–25 vs t034: 08–16–02–25–02–25–34–24–25) (https://spa.ridom.de/spatypes.shtml). Himsworth et al. evidenced shared MRSA lineages in human, livestock and rats, suggesting that rats can be a source of human and livestock infection [15]. In Austria, MRSA CC398 has been reported in livestock, companion animals [45] and human patients [46].

Himsworth et al. estimated the prevalence of MRSP in urban brown rats from Vancouver, Canada, to be 2.1% (5/237) which is close to the prevalence detected here (1.6%) [16]. The authors discussed the possibility of MRSP transmission between dogs and rats. 

We identified one *mecC*-positive MR *S. xylosus* (*mecA*-negative). This isolate was recently described in Loncaric et al. in a 5-year survey of *mecC*-positive coagulase-negative staphylococci from 767 wild animals in Austria and was the first report of *mecC*-positive MR *S. xylosus* in brown rat [47]. 

Although the interaction between urban wildlife reservoirs of AMR and human health risk remains unclear, the overall prevalence of AMR we observed in the sampled rats is of concern. Several rats colonised with multidrug-resistant isolates, including one carbapenem-resistant *En. xiangfangensis* ST114, were captured at Karlsplatz in a neglected garden used by homeless people as a dormitory in summer. This particular situation enhances the risk of spillover of antimicrobial-resistant bacteria. In cities, homeless and marginalised people are the most vulnerable to rodent-borne diseases [48], therefore rodent control remains an important priority for urban health.

We did not find any significant impact of the place of capture or the sex and age (approximated by body weight and length) of the rats on AMR occurrence or on the intensity of co-colonisation. Notably, rat Enterobacteriaceae and staphylococcal strains isolated from two urban sites located ca 3 km apart shared similar AMR profiles. These findings support the hypothesis that rats potentially acquire antimicrobial-resistant bacteria from the environment (including food waste) and suggest that they may act as a reservoir of AMR in cities. Nevertheless, proving rat-to-human transmission is highly challenging. Twelve animals carried more than one resistant isolates, which indicates a potential role of urban rats as melting pot for horizontal gene transfer between bacterial species acquired from different sources at different times [10]. In the wastewater ecosystem, rats are exposed to AMR through direct contact with antimicrobial-resistant bacteria but also with mobile genetic elements, such as bacteriophages or plasmids, that carry AMR genes [10,42,49].

The principal limitations of our study reside in the low sample size (n = 62) and number of sites investigated (n = 2). A greater sample size would have increased the accuracy of the prevalence estimation and the power of the statistical analysis, therefore providing a more detailed picture of the epizootiology of AMR in urban rats. A greater number of sites would have provided information on the spatial variability and environmental risk factors of AMR in the city. Furthermore, data on antimicrobial-resistant isolates from domestic animals and human patients in Vienna would have given a more complete overview of the epidemiological situation. 

## Conclusion

We demonstrate that urban rats are potential spreaders of β-lactamase-producing Enterobacteriaceae, including the *En. cloacae* pandemic ST114 clone, and of MRS, which are considered a major risk to global public health. Because rats are ubiquitous in cities, are in contact with sewage effluents, have a generalist and opportunistic diet, interact frequently with human wastes and have a small home range [8], they are a good candidate sentinel species to detect fine-scale variation in the distribution and sources of AMR [50]. Our results stress the importance of urban wildlife species, such as brown rats, as bio-indicators for AMR surveillance programmes in urban ecosystems. This study underlines the importance of One Health approaches in the global context of AMR.

## Note

*This designation is without prejudice to positions on status, and is in line with United Nations Security Council Resolution 1244/99 and the International Court of Justice Opinion on the Kosovo Declaration of Independence.

## Supplementary Data

12006000 Supplement
