# Supplementary material for: Urban brown rats (Rattus norvegicus) as possible source of multidrug-resistant Enterobacteriaceae and meticillin-resistant Staphylococcus spp., Vienna, Austria, 2016 and 2017
Source: Euro Surveill. 2019 Aug 8;24(32):1900149. doi: 10.2807/1560-7917.ES.2019.24.32.1900149 (PMC6693289; doi:10.2807/1560-7917.ES.2019.24.32.1900149)
Supplement: Supplement [file 1900149_DESVARS-LARRIVE_Supplement.pdf]

Urban Brown Rats (*Rattus norvegicus*) as Possible Source of Multidrug-Resistant *Enterobacteriaceae* and Methicillin-Resistant *Staphylococcus* spp., Vienna, Austria, 2016/17

Supplementary Materials

This supplementary material is hosted by *Eurosurveillance* as supporting information alongside the article "Urban Brown Rats (*Rattus norvegicus*) as Possible Source of Multidrug-Resistant *Enterobacteriaceae* and Methicillin-Resistant *Staphylococcus* spp., Vienna, Austria, 2016/17" on behalf of the authors who remain responsible for the accuracy and appropriateness of the content. The same standards for ethics, copyright, attributions and permissions as for the article apply. *Eurosurveillance* is not responsible for the maintenance of any links or email addresses provided therein.

Supplementary Figure S1. **Spatial distribution of the captured brown rats (*Rattus norvegicus*) and antimicrobial resistant isolates in Karlsplatz (S1A) and Danube Canal (S1B), September 13, 2016 - June 6, 2017, Vienna, Austria**

Maps were built using QGIS 3.4.5 (QGIS Development Team, 2018). Basemaps: Orthofoto 2016 Wien (Open Data Österreich, https://www.data.gv.at/)

Figure S1A. Karlsplatz, Vienna, Austria

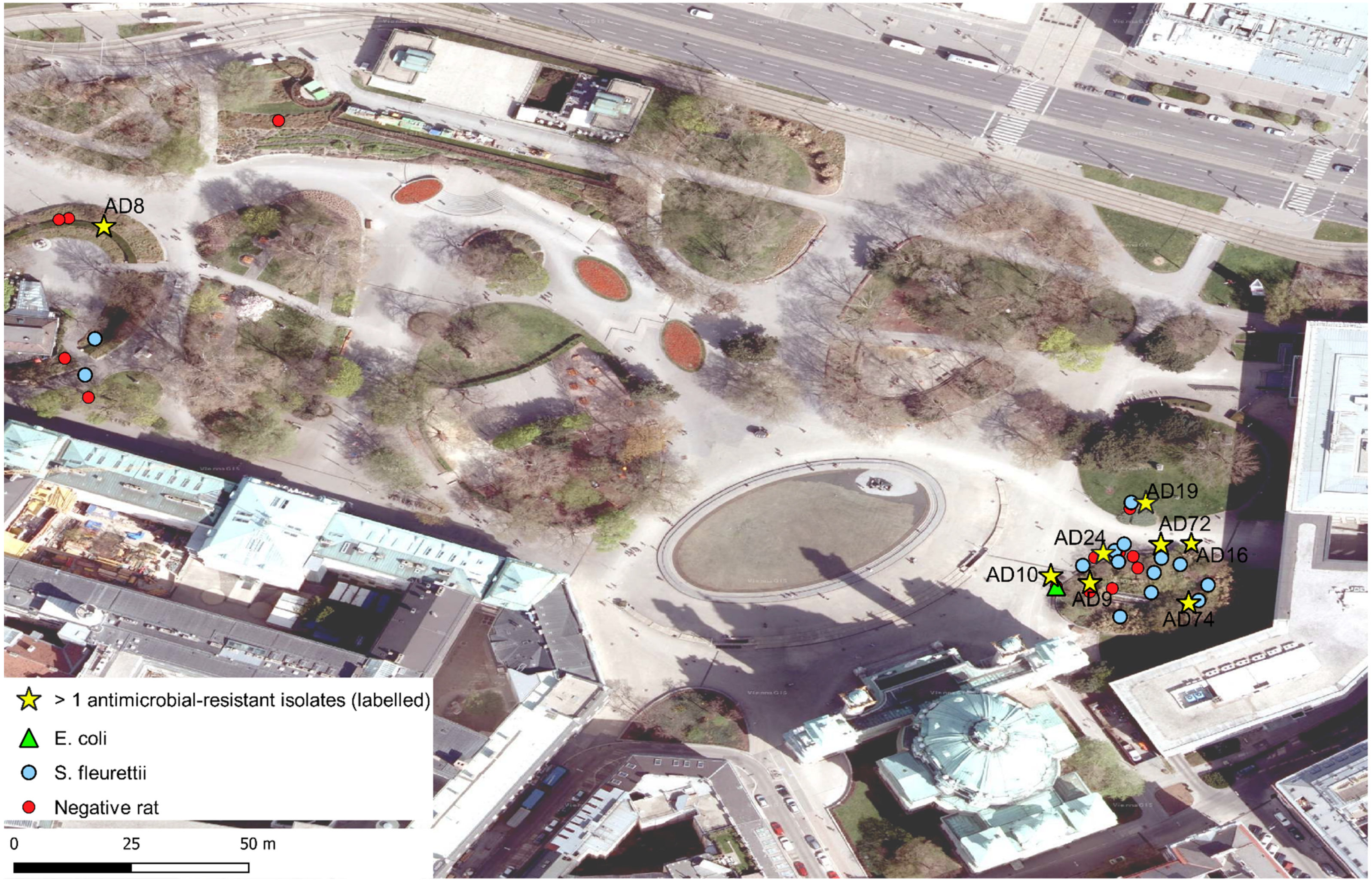

Figure S1B. Danube Canal, Vienna, Austria

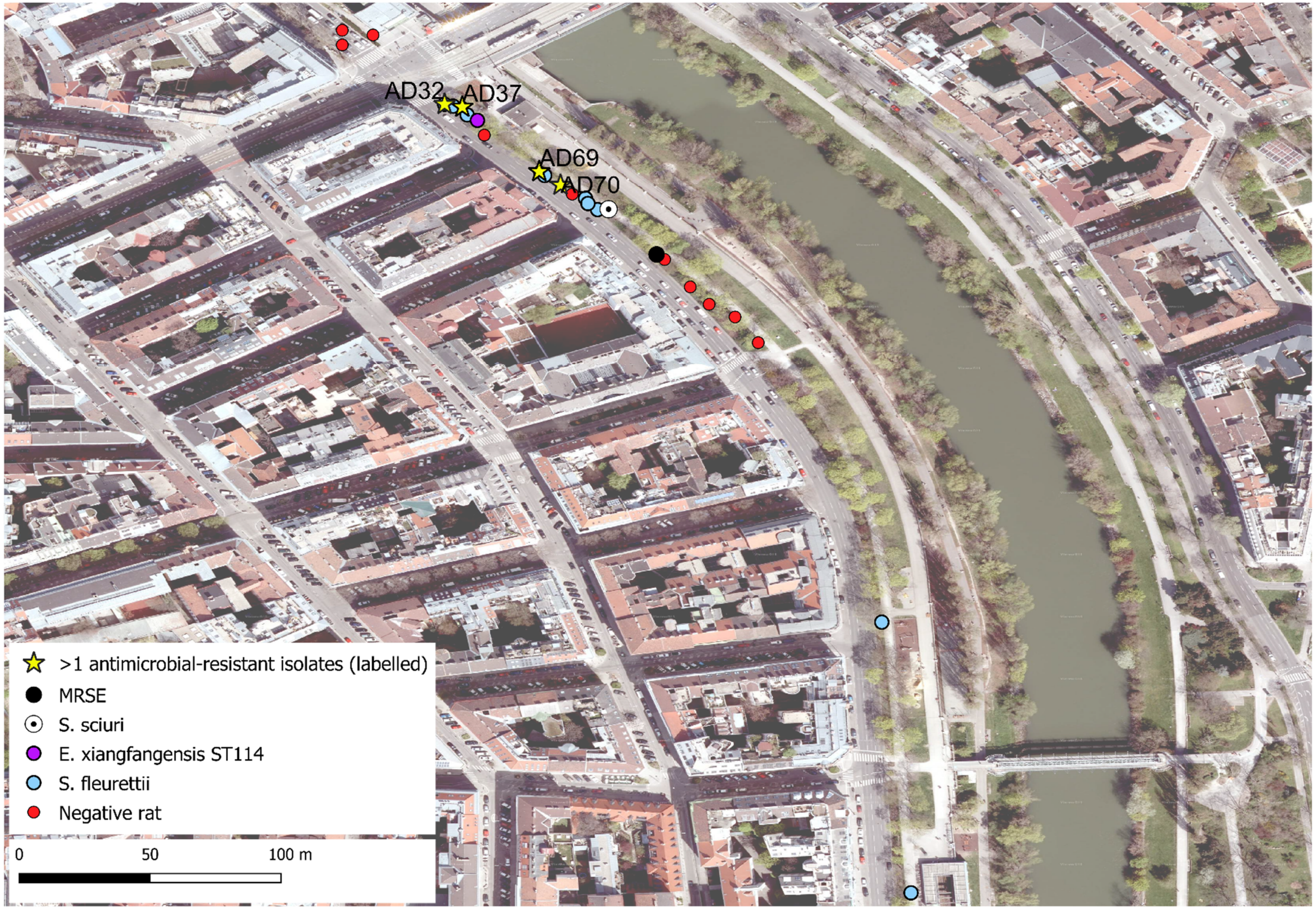

Supplementary Table S1. Summary characteristics of the brown rats (*Rattus norvegicus*) captured, September 13, 2016 - June 6, 2017, Vienna, Austria

Rat ID	Place	Date of capture	Latitude (D.D.)	Longitude (D.D.)	Elevation (m)	Sex	Body mass (g)	Length nose-anus (mm)	Length anus-tip tail (mm)	*Enterobacteriaceae* isolates (no.)	*Staphylococcus* spp. isolates (no.)
AD1	Karlsplatz	2016 Sep 13	48.19928	16.36938	135.7	male	264.9	229	174		
AD2	Karlsplatz	2016 Sep 13	48.20008	16.36992	188.5	male	185.3	191	163		
AD3	Karlsplatz	2016 Sep 13	48.1998	16.36932	174.4	female	65.7	138	128		
AD4	Karlsplatz	2016 Sep 13	48.19979	16.36929	173.9	female	65.3	143	125		
AD5	Karlsplatz	2016 Sep 20	48.19939	16.36931	166.4	male	269.7	232	194		
AD6	Karlsplatz	2016 Sep 20	48.19945	16.36939	166.7	male	276.5	223	186		*S. fleurettii*
AD7	Karlsplatz	2016 Sep 20	48.19935	16.36937	166.4	male	353.2	239	210		*S. fleurettii*
AD8	Karlsplatz	2016 Sep 20	48.19971	16.36931	168.8	female	334.3	238	221	*E. coli*	*S. fleurettii*
AD9	Karlsplatz	2016 Sep 27	48.1988	16.37246	181.3	female	209.8	214	183		*S. fleurettii*, MRSP
AD10	Karlsplatz	2016 Sep 27	48.19879	16.3723	180.4	female	352.1	239	250	*E. coli* (2)	*S. fleurettii*, *S. xylosus*
AD11	Karlsplatz	2016 Sep 27	48.19878	16.37243	179.9	female	76.6	143	130		*S. fleurettii*
AD12	Karlsplatz	2016 Sep 27	48.19879	16.37238	179.2	female	204.3	208	172		
AD13	Karlsplatz	2016 Sep 27	48.19884	16.37232	179.7	female	102.5	155	148		*S. fleurettii*
AD14	Karlsplatz	2016 Oct 11	48.19891	16.37234	132.5	female	42.1	119	111		
AD15	Karlsplatz	2016 Oct 11	48.19893	16.37233	131.8	female	38.3	107	91		*S. fleurettii*
AD16	Karlsplatz	2016 Oct 11	48.19894	16.37259	115.7	male	118.8	180.1	160		*S. fleurettii* (2)
AD17	Karlsplatz	2016 Oct 11	48.19886	16.3724	126.3	male	274.1	221	205		*S. fleurettii*
AD18	Karlsplatz	2016 Oct 11	48.19883	16.37233	128.0	female	238.9	206	186		
AD19	Karlsplatz	2016 Oct 11	48.19893	16.37237	133.7	male	46.6	121	111	*E. coli*	*S. sciuri*
AD20	Karlsplatz	2016 Oct 11	48.19875	16.37247	129.4	female	47.0	155	102		
AD21	Karlsplatz	2016 Oct 11	48.19877	16.37228	122.0	male	375.8	251	213	*E. coli*	
AD22	Karlsplatz	2016 Oct 11	48.19882	16.37226	121.0	male	242.2	288	187		
AD23	Karlsplatz	2016 Oct 11	48.19882	16.37245	123.9	male	351.0	242	204		*S. fleurettii*
AD24	Karlsplatz	2016 Oct 11	48.19877	16.37237	116.0	male	424.7	262	218	*E. coli*	*S. fleurettii*

AD25	Karlsplatz	2016 Oct 11	48.19879	16.37235	114.3	female	305.0	216	196		*S. fleurettii*
AD26	Karlsplatz	2016 Oct 18	48.19874	16.37227	114.0	female	33.9	111	99		
AD27	Karlsplatz	2016 Oct 18	48.19879	16.37241	111.9	male	40.9	102	95		*S. fleurettii*
AD28	Karlsplatz	2016 Oct 18	48.19881	16.37233	112.6	female	28.5	99	91		*S. fleurettii*
AD29	Karlsplatz	2016 Oct 18	48.19873	16.37231	89.0	female	30.1	100	100		
AD30	Karlsplatz	2016 Oct 18	48.19885	16.3723	77.8	male	32.1	105	102		*S. fleurettii*
AD31	Danube Canal	2017 Mar 6	48.22627	16.36576	168.6	male	381.5	249	194		*S. fleurettii*
AD32	Danube Canal	2017 Mar 6	48.22681	16.36497	177.2	female	408.9	240	195	*E. coli*	*S. fleurettii*
AD33	Danube Canal	2017 Mar 6	48.22681	16.36497	177.2	male	277.9	224	178		*S. fleurettii*
AD34	Danube Canal	2017 Mar 7	48.22673	16.36497	168.4	female	153.5	188	165		*S. fleurettii*
AD35	Danube Canal	2017 Mar 7	48.22629	16.36569	166.9	male	100.5	152	125		*S. fleurettii*
AD36	Danube Canal	2017 Mar 7	48.22673	16.36497	167.4	male	162.4	188	163		*S. fleurettii*
AD37	Danube Canal	2017 Mar 7	48.2268	16.36506	177.2	female	209.7	194	172		*S. fleurettii* (2)
AD38	Danube Canal	2017 Mar 7	48.22265	16.36738	167.6	male	211.8	214	184		*S. fleurettii*
AD39	Danube Canal	2017 Mar 7	48.22414	16.36715	169.1	female	211.4	190	190		*S. fleurettii*
AD50	Danube Canal	2017 Mar 21	48.22627	16.36582	153.0	female	152.9	180	155		*S. sciuri*
AD51	Danube Canal	2017 Mar 28	48.22632	16.36561	162.8	male	44.8	113	100		
AD52	Danube Canal	2017 Mar 28	48.2263	16.36571	163.3	male	45.8	122	115		*S. fleurettii*
AD53	Danube Canal	2017 Mar 28	48.22661	16.36519	159.0	female	319.4	206	226		
AD54	Danube Canal	2017 Mar 28	48.22669	16.36506	158.5	male	392.3	237	157	*E. xiangfangensis*	
AD59	Karlsplatz	2017 Apr 11	48.19875	16.37244	159.2	female	134.2	169	149		*S. fleurettii*
AD60	Danube Canal	2017 Apr 12	48.22587	16.36624	174.6	female	73.1	132	129		
AD61	Danube Canal	2017 Apr 12	48.22644	16.36549	184.2	female	49.0	123	113		*S. fleurettii*
AD62	Danube Canal	2017 Apr 12	48.22575	16.36629	174.3	male	80.0	142	137		
AD63	Danube Canal	2017 Apr 12	48.22572	16.36647	175.1	female	70.7	138	128		
AD64	Danube Canal	2017 Apr 12	48.22601	16.3661	175.4	female	303.2	219	172		
AD65	Danube Canal	2017 May 2	48.22717	16.36451	186.0	male	64.1	138	131		
AD66	Danube Canal	2017 May 2	48.22717	16.36461	184.9	male	61.5	130	116		
AD67	Danube Canal	2017 May 2	48.22558	16.36659	180.9	male	94.2	158	151		
AD68	Danube Canal	2017 May 2	48.22596	16.36605	179.1	male	296.5	223	200		MRSE

AD69	Danube Canal	2017 May 2	48.22646	16.36546	183.6	female	121.8	153	148		*S. fleurettii* (2)
AD70	Danube Canal	2017 May 2	48.22637	16.36555	183.0	male	120.4	157	142		*S. fleurettii*, MRSA
AD71	Karlsplatz	2017 May 3	48.19881	16.37248	194.0	male	210.9	214	176		*S. fleurettii*
AD72	Karlsplatz	2017 May 3	48.19876	16.37251	191.0	male	90.3	160	136	*E. xiangfangensis*	*S. fleurettii*
AD73	Danube Canal	2017 May 23	48.22719	16.36445	187.7	male	283.7	225	180		
AD74	Karlsplatz	2017 Jun 6	48.19872	16.37246	175.8	female	55.6	132	126	*E. coli*	*S. fleurettii*, MRSH
AD75	Karlsplatz	2017 Jun 6	48.19872	16.37246	175.8	male	201.7	202	181		
AD76	Karlsplatz	2017 Jun 6	48.19871	16.37235	176.1	male	292.7	228	191		*S. fleurettii*
